# Dynamics of the social construction of knowledge: an empirical study of Zhihu in China

**DOI:** 10.1140/epjds/s13688-022-00346-6

**Published:** 2022-06-04

**Authors:** Lun Zhang, Yong-Ning Li, Tai-Quan Peng, Ye Wu

**Affiliations:** 1 School of Arts and Communication, Beijing Normal University, Beijing, 100875 China; 2 School of Systems Science, Beijing Normal University, Beijing, 100875 China; 3 Department of Communication, Michigan State University, East Lansing, MI 48824 United States; 4 Computational Communication Research Center, Beijing Normal University, Zhuhai, 519087 China

**Keywords:** Mass collaboration, Marketplace of ideas, Knowledge construction

## Abstract

This study examined how the knowledge market promotes knowledge construction on question-and-answer (Q&A) websites. Data were collected from Zhihu, one of the largest Q&A sites in China. Hierarchical linear modeling was employed to estimate the dynamics of information accumulation, that is, the provision of informative content as factual construction. By employing information accumulation as the objective measure of knowledge construction, we determined that online knowledge construction was facilitated by a competitive marketplace of ideas. In addition, participation, temporal, and discourse features affected the dynamics of information accumulation. In specific, active users contributed significantly less to information accumulation than did ordinary users. Information accumulation shows a naturally decaying process represented as a function of answer order. The time interval between answers at the two preceding time points reduced the informativeness of answers at the subsequent time point. Answers with a higher readability score reduced the informativeness of subsequent answers. The results indicate that knowledge construction on Q&A sites unfolds as a process of mass collaboration among users.

**Supplementary Information:**

The online version contains supplementary material available at 10.1140/epjds/s13688-022-00346-6.

## Introduction

The structure of material commodity markets affects production. In contrast to competitive markets, monopolistic markets can determine the elasticity of demand and the price of a product [1]. In the marketplace of ideas, knowledge production is affected by the structure of an idea market. Whether the knowledge production process is more efficient in a competitive or monopolistic marketplace of ideas remains unclear. In a competitive marketplace of ideas, knowledge production is a social process in which the contributions of many participants are assembled through negotiation [2]. In a monopolistic marketplace of ideas, a few elite users dominate knowledge construction. A very small proportion of elite users, rather than a large crowd of average users [3], make substantial contributions to the advancement of knowledge.

Question-and-answer (Q&A) websites function as an online marketplace of ideas in which knowledge is constructed [4]. In these highly structured but open environments, freely editable content enhances the plurality of viewpoints in the knowledge construction process [5]. As a Q&A site, Zhihu.com is an example of a large-scale platform for knowledge construction. Zhihu is designed to allow people to ask and answer questions on a broad range of topics. Similar to Quora and StackOverflow, questions on Zhihu are resolved by user-generated answers. The question–answer threads on Zhihu are well-defined and easily identifiable artifacts [6]. A question–answer thread is a hierarchically organized collection of messages, with an initial answer to the original question and with subsequent messages written as answers to earlier messages [6] (please see the Supplementary Information for a sample page of Zhihu). Users can also post comments and reply to one another under each answer. This study used Zhihu to examine how the marketplace of ideas affects the dynamics of online knowledge construction. In particular, this study investigated whether online knowledge construction is better facilitated in a competitive marketplace of ideas or a monopolistic marketplace of ideas.

## Concepts, theoretical framework, and hypotheses

### Evaluating knowledge construction

Information accumulation is a key criterion of the success of the knowledge construction process on Q&A sites [7]. Knowledge construction on Q&A sites creates new ideas, explanations, and theories that help the members of a community understand the world [8–10]. According to Latour and Woolgar [11], information is accumulated in the knowledge construction process through the negotiation of new content among knowledge contributors. On Q&A sites, the provision of informative content follows an ordered progression of factual construction over time [5, 12].

However, information accumulation has been widely ignored in evaluations of knowledge construction on Q&A sites, especially Chinese Q&A sites. Studies have examined the information listed on Q&A sites by employing measures such as accuracy; completeness; article length; information richness; and the number of references, headings, and functional links [12], but few of them have employed the criterion of information accumulation. Therefore, the first objective of this study was to use information accumulation as an objective measure to examine the process of online knowledge construction in the context of Chinese Q&A sites, taking Zhihu as a case study.

### Marketplace of ideas

The marketplace of ideas, a concept underlying the notion of freedom of expression, is based on an analogy with the economic concept of a free market. With regard to Q&A sites, the marketplace of ideas refers to the act of discussion among distributed contributors. Q&A sites have become an ideal manifestation of the marketplace of ideas. Q&A sites embrace the tenets of marketplace theory, namely (1) that ideas should be in competition; (2) that the public at large is the best provider of ideas; and (3) that exposure to contradictory points of view has value in the search for truth [13]. On Q&A sites, users compete with one another to dominate the discussion, demonstrating the competition of ideas on the open market, which is the key component of marketplace theory. Q&A sites also reduce barriers to entry in the marketplace of ideas because all opinions can be freely expressed, with the open exchange of ideas. Furthermore, an essential function of Q&A sites is the ability of users to read and reply multiple answers to a question and be exposed to conflicting points of view; this can promote the diversity of opinions. These characteristics make Q&A sites ideal online communities to which marketplace theory can be applied to study knowledge construction [13].

The second objective of this study was to investigate the contribution of the marketplace of ideas to information accumulation on Q&A sites. Q&A sites can be generally classified as a competitive marketplace of ideas or a monopolistic marketplace of ideas [14]. In a competitive marketplace of ideas, the sharing of contributor viewpoints can be understood as an exercise undertaken to resolve differences of opinion [5]. Answers on Q&A sites reveal competition among ideas. In addition, in a more competitive marketplace of ideas, free expression is self-correcting [15]. A consensus emerges from competition among ideas in a free, transparent public discourse, and ideas and ideologies are winnowed on the basis of their superiority or inferiority and their acceptance by the user population.

By contrast, in a monopolistic marketplace of ideas, the flow of information is controlled. The dominant viewpoints monopolizes the marketplace and drives the other information out, thus excluding rival opinions [16]. In a Q&A site that operates as a monopolistic marketplace of ideas, the critical thinking of knowledge contributors is suppressed. Therefore, in a monopolistic marketplace of ideas, less-diverse viewpoints are disseminated to the public, thus generating less new knowledge [17]. Therefore, we proposed the following hypothesis:

#### Hypothesis 1

*A more competitive marketplace of ideas contributes more to information accumulation than a more monopolistic marketplace of ideas does*.

### Participation features

On the basis of the study by Latour and Woolgar [11] and other studies on the collaborative construction of knowledge [2, 18], the present study further included participation features, temporal features, and discourse features as explanatory variables to explain the knowledge construction process.

Q&A sites promote social participation, which is pivotal to facilitating knowledge contributions because it promotes the development of social cohesion and a sense of belonging [8–10]. According to Gunawardena *et al*. [2], participation features determine the key stages of knowledge construction, which are the discovery and exploration of dissonance or inconsistency among ideas, concepts, or statements; the negotiation of meaning and co-construction of knowledge; and the testing and modification of the proposed synthesis or co-construction process. On Q&A sites, participation features may affect users’ motivation to contribute knowledge and the manner in which they share their existing knowledge.

#### Presence of active users

Participation feature in current study refers to the presence of active users. In online knowledge construction, most active users make crucial contributions to information accumulation. A widely observed phenomenon in online communication is the “90–9–1 rule.” This refers to the fact that in most online communities, 90% of users never contribute but only consume content, 9% of users have a little contribution, and 1% of users account for almost all of the contributions [19–21]. Studies reported the presence of uneven contributions in online collaborative settings [22, 23]. For example, Serrano *et al*. [24] proposed a ratio metric to calculate the inequality of participation in Wikipedia: the quotient between the number of contributions from the top 10% contributors and the number of contributions from the other 90% users. They observe a power-law in the distribution of contributions. Movshovitz-Attias *et al*. highlighted the importance of the contribution of active users to the answers posted on StackOverflow [25]. Users with high numbers of contributions were usually the first responders to questions. If the questions remain unanswered, they are then answered by users with fewer number of contributions. Accordingly, we posited the following hypothesis:

##### Hypothesis 2

*Unlike ordinary users*, *active users promote information accumulation*.

### Temporal patterns of knowledge construction on Q&A sites

Temporal features refer to the time interval between answer posts and the order of answers. Temporal features result in the construction of knowledge in a hidden and unintentional manner [26, 27]. First, online knowledge creation is not a linear function of time. A marked reduction in the interval between answer posts on a site (i.e., a spike in answers posted in a given period) can serve as a proxy for a peak in online collaboration [28]. Q&A sites are web environments in which content is stored and managed collaboratively. They are also platforms on which users can showcase their knowledge and expertise to peers or potential recruiters [29]. Surges in posting behavior on Q&A sites can promote emergent collaboration [30] in terms of improvisation [31], heedful interrelating [32], privileging expertise [33], and knowledge shaping [34]. Users may provide more responses in a short period of time under external or internal triggers [30], such as a controversial topic, the presentation of biased information, or some form of vandalism (i.e., editing deliberately intended to defeat or obstruct other contributions). In this regard, a short time interval between answer posts indicates the burstiness of knowledge construction. Burstiness in this context is characterized by users collaboratively engaging in the knowledge construction process in a short period of time, during which knowledge is accumulated. Anderson *et al*. [35] observed that the majority of answers on Q&A sites are posted within a day after the question is asked. In addition, the longer a question remains unanswered, the lower the likelihood is that a satisfactory answer will be provided. Therefore, we postulated the following hypothesis:

#### Hypothesis 3

*The time interval between answer posts affects the dynamics of knowledge construction*. *The shorter the time interval between answer posts*, *the higher the level of information accumulation*.

Second, information accumulation is a naturally decaying process. The amount of new information decreases along with the increase of the order of answers, because multiple users offer the same information or refer to the same source for answering a single question. The first answers tend to provide novel information, whereas answers provided at the end of the thread tend to be less informative. Anderson *et al*. [35] reported that on StackOverflow, the most informative answers usually appear earlier in the sequence of responses to a question. Therefore, information accumulation decreases with the order of answers because of the repeated referral to similar information. We presented the following hypothesis:

#### Hypothesis 4

*Information accumulation is a naturally decaying process represented as a function of answer order*.

### Discourse features

Discourse features refer to sentiment and readability. As noted by Latour and Woolgar [11], in online knowledge construction, the discourse is subjected to meaning negotiation. Information that conveys sentiment [36] and is easy to understand is more likely to stimulate online engagement.

#### Sentiment

In this study, we employed sentiment and readability as discourse factors. Sentiment can affect users’ knowledge contributions to a platform. Q&A sites facilitate numerous types of interactions. On these online collaboration platforms, members may be driven to answer questions for conveying their sentiment appropriately through text [37]. From the perspective of interpersonal communication, individuals’ sentiment toward a topic represents the atmosphere of the discussion on that topic. Expressions of gratitude and reciprocity are essential for developing trust and empathy among users, which increases their willingness to collaborate and the acquisition of answers on Q&A sites [38]. A study by Jiao *et al*. determined that replies that express positive sentiment (e.g., “that’s a great summary”) can lead to further communication [39]. By contrast, replies that convey negative sentiment (e.g., “I am so confused” and “Shut up”) are more likely to end a conversation [40].

Sentiment also indicates knowledge contributors’ interest in a topic. Positive sentiment of a topic on Q&A sites correspond to greater interest in that topic [41, 42]. If a group of people have positive sentiment for a topic, they are more likely to focus on that topic and contribute to the platform [43]. Accordingly, we advanced the following hypothesis:

##### Hypothesis 5

*The positive sentiment of an online discussion leads to the accumulation of information*, *whereas the negative sentiment of an online discussion reduces the accumulation of information*.

#### Readability

Readability refers to the textual comprehensibility of a discourse [44]. On Q&A sites, readability represents the ease with which a piece of text can be understood [45]. Journal articles written for expert audiences and answers on Q&A sites written for nonexpert readers differ substantially in their readability [46]. Knowledge constructed by expert users with extensive domain-specific knowledge is often difficult to understand because such users assume that their readers are fellow experts who can easily comprehend abstruse scientific text [47]. Online knowledge construction on Q&A sites entails the reformulation of professional content for the non-specialist public through linguistic means, which supports the popularization of this content on the sites [48]. Knowledge constructed for general users who have little or no prior knowledge of a given topic or field must be easily understandable. Higher readability can increase the probability that the text conveys the correct information with minimal reading effort [45].

The readability of text on Q&A sites may affect the dynamics of knowledge construction on such sites [45]. The use of jargon-free language may increase the accessibility and acceptability of scientific knowledge to the non-specialist public. Readable language improves the reception of a post and attracts a greater number of general users, encouraging them to join the discussion. Therefore, we proposed the following hypothesis:

##### Hypothesis 6

*An online discussion with high readability facilitates the accumulation of information*.

On the basis of the findings of Harper *et al*. [49] and Kim *et al*. [50], we classified questions posted on Q&A sites into three types: opinion based, experience based, and fact based. The question type represents the style of response required for the question. Opinion-based questions require others’ thoughts on a topic of general interest; these questions do not have a correct answer. Experience-based questions require that respondent’s share their experiences. Fact-based questions require the provision of objective information.

In addition, we employed the number of answers to each question as an additional control variable. The number of answers indicates the popularity of a question. We controlled for the number of answers to examine the effects of the marketplace type (i.e., competitive or monopolistic) on information accumulation.

## Methods

### Data collection and questions filtering

Data for this study were collected from Zhihu, a widely used Q&A site in China. Zhihu is an example of a large-scale, volunteer-led collaborative project to construct knowledge [15]. Zhihu was launched in 2011, and by 2019, the number of registered users had exceeded 220 million.^1^

**Figure 1:**
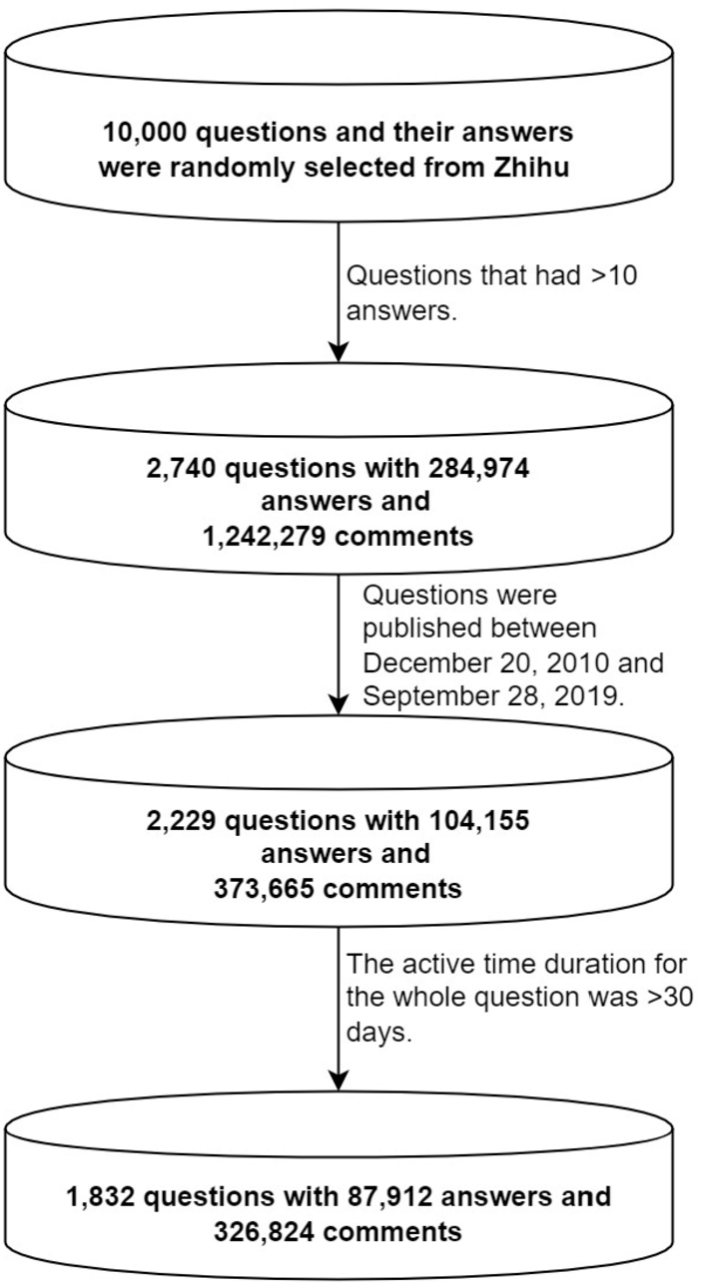
Data collection and filtering

Large-scale datasets contain a high number of observations, which makes rejecting the null hypothesis at the selected alpha level considerably easier ($p < 0.05$ in this study)[51]. Therefore, we randomly selected 10,000 questions and their answers from a list of retrieved questions ($N = 1\text{,}600\text{,}000$) from Zhihu. Figure 1 illustrates the data collection process. We filtered the questions using the following criteria. First, only questions that had more than 10 answers were retained. Second, only questions that were published between December 20, 2010 (Zhihu’s launch date), and September 28, 2019, were included. Third, only questions that were open to answers for more than 30 days were retained. The final sample comprised 87,912 answer records nested within 1832 questions.

### Measurements

**Figure 2:**
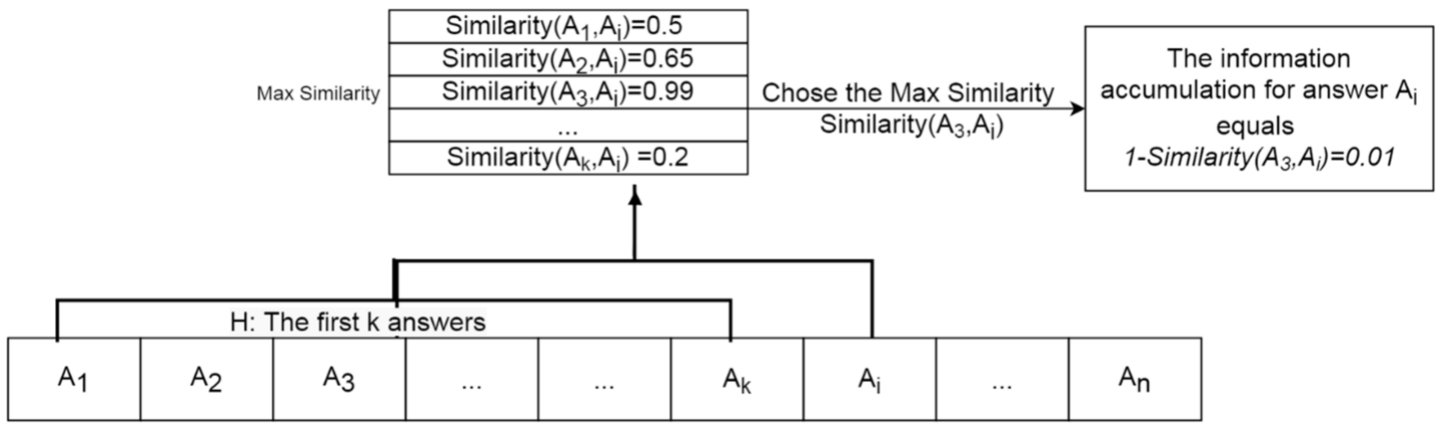
Schematic of information accumulation measurement

Information accumulation was measured at the answer level. For each question thread, we determined the dynamics of information accumulation by employing the computational approach proposed by Qi, Zhang, and Manning [52]. For a given question *Q*, *H* represents its historical answer sequence, which contains *k* answers before $A_{i}$, where $A_{i}$ is the *i*th answer of *Q*, as expressed by the following equation (Fig. 2):

1 $$ H= \{ A_{1}, A_{2}, A_{3},\ldots , A_{k} \}. $$

The information accumulation of answer $A_{i}$ represents the amount of new information the *i*th answer holds relative to the content of previous replies. The assumption is that the more the amount of new information $A_{i}$ holds, the higher the likelihood is that it would include words not used in preceding answers. To quantify the information accumulation of $A_{i}$, we first estimated the pairwise lexical similarity between $A_{i}$ and all other preceding answers $A_{k}$ in *H* ($1 < k < i < n$, where *n* is the total number of answers). For example, for a question with four answers, the similarity of the fourth answer to the preceding three answers (i.e., the similarity between the fourth and third, fourth and second, and fourth and first answers) was estimated.

The Doc2vec algorithm [53] was employed to estimate the similarity between $A_{i}$ and all preceding answers listed in a historical answer sequence *H*. An extension of Word2vec, which extends the learning of word embeddings from words to word sequences, Doc2vec converts a document to a distribution over words, which can be used in pairwise comparisons of the document similarity. The cosine similarity score ranges from −1 to 1. Because negative values of the similarity score are meaningless, we drop the observations with negative similarity scores from the analysis and constrained the similarity score from 0 to 1[54]. A similarity score of 0 indicates the absence of similarity between $A_{i}$ and $A_{k}$. A similarity score of 1 indicates that $A_{i}$ is a replicate of the preceding answer $A_{k}$. Appendix provides further validation of information accumulation (see Additional file 1).

The estimated similarity scores were used to calculate the information accumulation of the *i*th answer by using the following formula (2):

2 $$ IA_{\mathrm{ans}} ( A_{i} ) =1- \max_{1< k< i} \text{Similarity} ( A_{i}, A_{k} ). $$

#### Measurement validation

Manual coding was performed to validate the computational measurement of information accumulation. Two coders were recruited to rate the information accumulation of answers to each of 400 randomly selected questions. They conducted a pairwise comparison of $A_{i}$ and each preceding answer in the sequence by answering the question “Does the given answer (i.e., $A_{i}$) provide new information?” with the possible responses of 1 (*yes*) and 0 (*no*) on a dichotomous scale. If any rating for a given answer $A_{i}$ was 0, the information accumulation of $A_{i}$ was given a final score of 0. This meant that $A_{i}$ failed to provide new information. Intercoder reliability was adequate, as indicated by Krippendorff’s alpha of 0.82.

The information accumulation of all 400 questions was estimated using our computational algorithms. The estimated score was classified as a binary variable, with scores greater than 0.5 recoded as 1 and scores lower than 0.5 recoded as 0. The correlation between the human-coded results and the computationally estimated results of the information accumulation of the 400 questions was 0.68, which demonstrated that our computational algorithm yielded results of information accumulation that were consistent with those of human assessments (please see Additional file 1 for further validation of the measurement).

#### Marketplace of ideas

The marketplace of ideas was assessed at the answer level. For each answer ($A_{i}$) nested within a question, we obtained a distribution of the answer length (the number of characters in each answer) and the number of comments left on each preceding answer.

The Gini coefficients for the answer length and the number of comments were used to assess the marketplace of ideas of $A_{i}$ before answer $A_{i}$ was provided. The Gini coefficient, an entropy measure of the equality of distribution, is a measure of statistical dispersion and is representative of inequality. Therefore, a low Gini coefficient indicates a competitive marketplace of ideas, which lacks dominant viewpoints, whereas a high Gini coefficient indicates a monopolistic marketplace of ideas for given questions.

The Gini coefficient of the answer length indicates the equality of the distribution of the knowledge provided by contributors in the construction stage. The distribution of the number of comments for answers indicates the preferences of other users. The Gini coefficient of the number of comments indicates equality in the distribution of users’ feedback. A Gini coefficient of 0 for the number of comments indicates an equal distribution; that is, all answers received the same number of comments. A Gini coefficient of 1 represents maximum inequality; that is, all comments were left under one answer, and the other answers received no comments.

#### Presence of active users

To determine the presence of active users, we calculated the answering frequency of all users on Zhihu. On the basis of the 90–9–1 rule, we defined highly active, active, and ordinary users as those whose answering frequencies ranked in the top 1%, top 2%–10%, and bottom 90%, respectively. Anonymous users, who have private accounts and cannot be identified in the platform, were classified in a separate category. The users on the platform are fully anonymous. We cannot tell if two messages in the same discussion by anonymous users are by the same person.

#### Sentiment

Sentiment was calculated as the intensity scores of neutral, positive, and negative sentiment for a given answer. We used Senta, an open-source sentiment classification system established by Baidu for Chinese text sentiment analysis. We employed the BiLSTM (i.e., Bidirectional Long Short Term Memory) model, which is a recurrent neural network model that accepts Chinese corpus as input and outputs sentiment polarity [55, 56]. A score of 0 represents negative sentiment, and a score of 1 represents positive sentiment.

#### Readability

Several readability metrics exist that can be used to quantify the readability of English-language text, such as Flesch–Kincaid readability test, Coleman–Liau index, and automated readability index [45]. In this study, we evaluated readability by using graded vocabulary lists in a Chinese proficiency test, namely Hanyu Shuiping Kaoshi [HSK], and by using English-language readability measures. The total number of words in the HSK lists is 23,760, which are divided into six levels.

Readability *R* corresponded to the combination of the average number of words per sentence and the average HSK grade per word [57] and was calculated as follows:

3 $$ \boldsymbol{R} = \mathbf{ln} \biggl( \frac{\boldsymbol{W}}{\boldsymbol{N}} \biggr) + \frac{\sum_{\boldsymbol{i} = \mathbf{1}}^{\boldsymbol{T}} \boldsymbol{Li}}{\boldsymbol{T}}, $$

where *W* represents the word count of a given answer, *N* represents the total number of sentences, *T* represents the total number of words assigned an HSK grade, and $L_{i}$ represents the score of the *i*th word assigned an HSK grade ($i \in [1, T]$). We scored words from HSK levels 1 to 6 as 1 to 6, respectively ($L_{i}\in [1,6]$). Readability is an inverse measure; the higher the readability score of a text, the higher the complexity of that text. We further provided an example to illustrate how to calculate readability in the Additional file 1.

We manually coded the questions into experience-based, opinion-based, and fact-based queries. Experience-based questions require the provision of information on the personal experience of respondents (e.g., “How is your experience with using Google Pay?”). Opinion-based questions inquire about respondents’ opinions (e.g., “Why is Lewis Hamilton so generally disliked?). Fact-based questions inquire about objective facts (e.g., “Is it possible to fly a rocket through Jupiter?”).

### Data analysis

We performed hierarchical linear modeling (HLM) by estimating the maximum likelihood across two levels (i.e., within-question knowledge contribution behavior and between-question differences in knowledge contribution). The within-question features (i.e., marketplace of ideas, participation features, discourse features, and temporal features) were postulated to be nested within the between-question factors (i.e., question types and number of answers) in influencing the dynamics of information accumulation. HLM is a useful method for analyzing longitudinal data, enabling researchers studying the dynamics of information accumulation over time to fit various advanced regression models into longitudinal datasets.

A lower-level (i.e., level 1) model presented the information accumulation for each question as a linear function of the marketplace of ideas, participation feature, temporal features, and discourse features. The parameters of the lower-level variables were considered dependent variables in the higher-level (i.e., level 2) model. Level 2 parameters were predicted using the question type and number of answers. To estimate the goodness of fit, we established a null model: a model with no level-1 or level-2 predictors. We then compared the reduction of the log-likelihood ratio (i.e., −2LL) of the null model with that of the full model (Table 1).

**Table 1:** HLM regression for information accumulation

	Model 1		Model 2	
	Estimates	Errors	Estimates	Errors
Fixed Effects				
Intercept	0.28	0.002	0.46	0.01
*Marketplace of Ideas*				
Gini-Answers Length			−0.11^∗∗∗^	0.01
Gini-Comments			−0.11^∗∗∗^	0.01
*Participation Features*				
Presence of Active Users^a^				
Highly Active Users			−0.01^∗∗∗^	0.003
Active Users			−0.01^∗∗∗^	0.002
Anonymous			0.001	0.001
*Temporal Features*				
Time-Interval between Answers^b^			−0.01^∗∗∗^	0.004
Order of Answers			−1.44^∗∗∗^	0.08
*Discourse Features*				
Sentiment			0.0002	0.001
Readability^c^			−0.01^∗∗^	0.003
*Control Variables*				
Question Type: Opinion^d,e^			0.003	0.004
Question Type: Fact^d,e^			−0.04^∗∗∗^	0.01
Number of Answers per question^b,d^			−0.004^∗^	0.002
*Random Effects*				
Intercept T_00_	0.07	0.16	0.07	0.15
*Model Fit Statistics*	37,451		39,705	

*Notes*. *** *p*<0.001; ** *p*<0.01; * *p*<0.05

^a^Highly active users are users whose answering frequencies rank in the top 1%. Active users are users whose answering frequencies rank in top 2%–10%. Ordinary users are users whose answering frequencies rank in bottom 90%. The comparison group is ordinary users.

^b^Variables are log transformed.

^c^Readability is an inverse measure. The higher the readability score of a text is, the higher the complexity of the text is.

^d^Variables at level 2 (i.e., the question level).

^e^The comparison group is Experience-based questions.

## Analytical results

### Dynamics of information accumulation among questions

**Figure 3:**
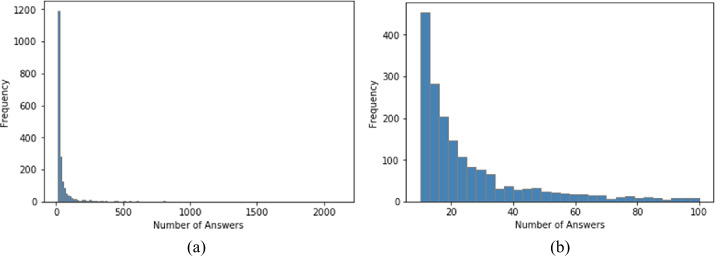
Distribution of the number of answers; (**a**) all answers; (**b**) truncated distribution of questions that had fewer than 100 answers

For all 1832 questions, the number of answers exhibited a typical skewed distribution, indicating that most questions received few answers, and that few questions received many answers. The number of answers for the 1,832 questions ranged from 10 to 2114. For 75% of the questions, the number of answers was <40. Figure 3(a) displays the truncated distribution of questions that had fewer than 100 answers.

**Figure 4:**
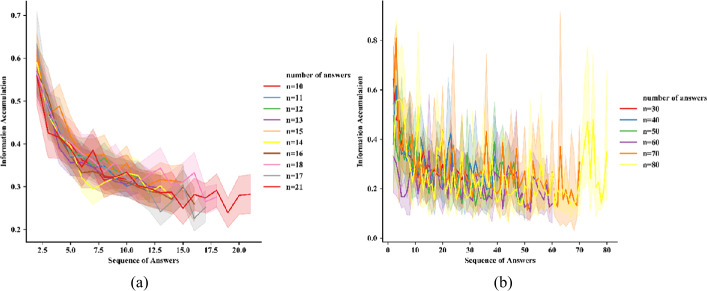
Dynamics of information accumulation. (**a**) questions receiving 10 to 21 answers; (**b**) questions receiving less than 80 answers

To represent the dynamics of information accumulation, we selected questions that had a typical number of answers. As shown in Fig. 4(a), we ranked the frequency of answers to all questions and selected questions receiving 10 to 21 answers (corresponding to the tenth rank of the number of answers). The dynamic of information accumulation with questions receiving less than 80 answers is shown in Fig. 4(b). In Fig. 4(a), the *x*-axis represents the order of answers (i.e., the *n*th answer), and the *y*-axis represents the mean value of information accumulation between the *n*th answer and the first $(n - 1)$th answer. The colors of the lines represent questions with different numbers of answers. For example, the blue line represents the dynamics of information accumulation for all questions with 10 answers. The shadow represents the 95% confidence interval.

All questions with different numbers of answers exhibited a similar pattern of information accumulation. The information accumulation of the ($n + 1$)th answer to the preceding answer (i.e., the *n*th answer) decreased rapidly during the early stage of knowledge construction; that is, the increase in informativeness tapered off rapidly over this period. With the completion of knowledge construction, the information accumulation of the ($n + 1$)th answer to the *n*th answer leveled off; no further information was accumulated at the end of the answer sequence. This finding is consistent with that of Keegan and Tan [30], who reported that subsequent answers provided on Wikipedia to COVID-19-related questions exhibited a consistent decrease in information novelty. This pattern indicated that the online knowledge construction process is completed through ongoing changes rather than sudden shifts.

### Factors affecting information accumulation

Table 1 lists the HLM results, with the features of questions (level 2) presented as aggregated variables and the characteristics of answers (level 1) presented as individual variables. To determine how well the model fit the data, a null model (i.e., model 1; that is, a model with no level-1 or level-2 predictors) was first estimated. Model 2 was a full model, also known as an intercept-as-outcome model. Model fit in HLM was determined using the log-likelihood ratio, a deviance statistic. A large log-likelihood ratio indicates an unfavorable model fit, and a small log-likelihood ratio indicates a favorable model fit. The model fit can be determined by comparing the deviances between the full and null models. The difference between the two deviance statistics follows a chi-square distribution, in which the degree of freedom equals the difference in the number of parameters estimated between the two models. If the $\chi ^{2}$ value is statistically significant, the model with a low deviance value has a significantly more favorable fit. The present model explained 47% of the variance, which is significantly more than that in a previous study investigating the individual information contribution of platform users [58].

Both within-question–level and between-question–level variables in the model significantly explained information accumulation (Table 1). The intraclass correlation coefficient (ICC) of the model was 0.18, indicating that approximately 18% of the variance in information accumulation could be explained by between-question differences. This ICC was greater than zero, suggesting that HLM regression was required to determine the fixed effects, as opposed to conventional methods such as analysis of variance [59].

Table 1 displays the HLM regression results. The effects of Gini coefficients of both the answer length ($\beta = -$0.11; $p < 0.001$) and the number of comments ($\beta = -$0.11; $p < 0.001$) in information accumulation were significantly negative. This indicates that the more unequal is the distribution of users’ viewpoints to a question, the lower the likelihood of information accumulation over time. For a given question, a more competitive marketplace of ideas contributes more to information accumulation than a more monopolistic marketplace of ideas does. Therefore, Hypothesis 1 was supported.

Highly active users (i.e., answering frequencies ranked in the top 1%) and active users (i.e., answering frequencies ranked in the top 2%–10%) contributed significantly less to information accumulation than did ordinary users (i.e., answering frequencies ranked in the bottom 90%). Therefore, Hypothesis 2 was not supported.

The time interval between answers at the two preceding time points (i.e., time *t* and time $t-1$) negatively affected the informativeness of answers at the subsequent time point ($\beta = -$0.01; $p < 0.001$). The longer the time interval between two answers was, the lower the informativeness of answers at the subsequent time point was. Therefore, Hypothesis 3 was supported.

The order of answers significantly predicted the dynamics of information accumulation ($\beta = -1.44$; $p< 0.001$), indicating that the knowledge construction process is a function of the answer sequence. Therefore, Hypothesis 4 was supported.

The sentiment of the online discussion exerted no effect on information accumulation. Therefore, Hypothesis 5 was not supported. This result is in line with that of Harper *et al*. [60], who observed that on Q&A sites, expressions of gratitude did not significantly predict answers to a question.

Answers with a higher readability score reduced the informativeness of subsequent answers ($\beta = -0.01$; $p < 0.01$). Because the readability value of text represents its complexity, the results indicate that the complexity level of the online discussion reduced the accumulation of information. Therefore, Hypothesis 6 was supported.

Regarding control variables, the number of answers was determined to negatively influence information accumulation ($\beta = -0.004$; $p < 0.05$). Information accumulation decreased with an increase in the number of answers (see Additional file 1 for the follow-up test of interaction effects between the number of answers and Gini coefficient). Regarding the question type, compared with experience-based questions, threads for fact-based questions ($\beta = -0.04$; $p < 0.001$) exhibited a lower level of information accumulation.

## Discussion

In this study, online knowledge construction was facilitated in a competitive marketplace of ideas by information accumulation. By contrast, the monopolization of the marketplace restricted the community’s ability to progress and innovate in terms of thoughts and ideas. Participation features, temporal features, and discourse features also affected the dynamics of information accumulation. The results demonstrated that Q&A sites are not only a large repository for knowledge but also a new field for information exchange and knowledge construction. The emergence of Q&A sites has transformed knowledge construction into a collaborative and thoroughly socialized process [61].

Most of the questions exhibited a similar pattern of information accumulation. New information accumulation in follow-up answers initially increased rapidly but then decreased gradually. The informativeness of answers decreased rapidly during the early stage of knowledge construction, and no additional information was accumulated at the end of the answer sequence. Temporal features, namely the interevent time and the order of answers, also affected the dynamics of information accumulation. A short between-answer interval increased information accumulation. This suggests that knowledge construction on Q&A sites is a dynamic process through which new information is continually accumulated.

The model factors indicate that online knowledge construction involves continual collaboration between users. Highly active users did not contribute substantially to information accumulation. Highly active users may be highly conforming and may demonstrate homogeneity in their language use on the platform. Therefore, knowledge construction on Q&A sites is not dependent on the contributions of such users despite their high number of contributions on the platforms. Instead, the knowledge construction process is characterized by limited contributions from a large number of less active users [62]. This result is consistent with the Ortega hypothesis, which states that a large pool of ordinary contributors is essential to a system’s overall functioning.

Our results indicate that the characteristics of the marketplace of ideas are major factors that drive information accumulation on Q&A sites. Knowledge is more likely to accumulate in a competitive marketplace of ideas than in a monopolistic marketplace of ideas. This conclusion is based on the observation that equal distributions of answer lengths and numbers of comments promoted information accumulation.

### Possible explanations for the facilitation of information accumulation by a competitive marketplace of ideas

We present a possible explanation for the facilitation of information accumulation in a competitive marketplace of ideas. Because a competitive marketplace of ideas lacks dominant viewpoints [2], it is characterized by disagreement and dissonance. The development of knowledge is triggered by individual cognitive disturbance, that is, the prolific resolution of socio-cognitive conflict [62]. Cognitive disturbances result from individuals’ realization that others’ cognitive schemata differ from theirs, as represented by disagreement and dissonance on the Q&A sites. Addressing the disturbances in a productive manner facilitates learning and collaborative knowledge construction. In the long term, this leads to innovation and information accumulation [62, 63].

### Perspective shift in research on knowledge construction

Theories and models have indicated that collaborative knowledge construction occurs at both intrapersonal and interpersonal levels. At the intrapersonal level, psychological and cognitive systems contribute to users’ knowledge construction and retrieval behaviors. These behaviors include the retrieval of knowledge from long-term memory [64], the elaboration of knowledge [65], and the externalization and internalization of knowledge. Knowledge contribution occurs through the internal processes of assimilation and accommodation. On Q&A sites, users refine and reassemble their knowledge within a certain domain. Personal knowledge must be logically and comprehensibly conveyed as an answer. This requires the in-depth processing and clarification of information [62]. Accordingly, knowledge construction is considered to be a product of users’ active participation in an online environment [66].

At the interpersonal level, knowledge contributors who utilize knowledge resources in the form of electronic artifacts constitute a community of individuals who share a common interest and are willing to share their ideas with other community members or to respond to their ideas. Peer facilitation techniques that increase knowledge contributions have been studied in the literature. Peer facilitation refers to online discussion facilitated by participants in knowledge contribution settings [67]. Peer facilitators have been demonstrated to be instrumental in shaping the discourse, and the use of peer facilitation results in effective online discussions [68, 69].

In sum, numerous studies have framed knowledge construction as either an individual-level or bilateral behavior. These framings do not holistically reflect the dynamic pattern of knowledge construction, and knowledge is erroneously considered to exist on its own rather than being a dynamic product on which users’ interactive sequences are based. This misperception can be attributed to the development of models using small data samples of Q&A sites, which cannot accurately model and theorize the dynamics of knowledge construction.

This study contributes to the literature on knowledge construction by shifting the focus from intrapersonal and interpersonal communication to the process of knowledge construction at the question–answer level. The sequence perspective emphasizes that no hidden correct knowledge awaits discovery. Knowledge is constructed when answers are crafted and circulated through an online community. The manner in which knowledge develops on Q&A sites indicates that knowledge is rarely purely individual and is never static [62, 70]. Rather, knowledge construction on Q&A sites is cumulative and depends on previous answers.

### Practical implications

The results correspond to a nonessentialist view of knowledge construction and contain practical implications for the management of Q&A sites. The contributions of less active platform users have been considerably underestimated. For example, Amazon mainly reward their top knowledge contributors. Q&A sites should reward users on the basis of the informativeness of their answers rather than on the basis of their answering frequency.

## Limitations

This study had several limitations. First, this study explored the dynamics of information accumulation in the context of a Q&A site. Collaborative knowledge construction varies subtly among different online knowledge sharing platforms. For example, Wikipedia is an open-source repository of reference knowledge to which users contribute knowledge by collaboratively writing and editing articles [12]. The premise that different online knowledge-sharing platforms share elements of knowledge construction is reasonable. Future research could generalize the results to other online knowledge sharing platforms.

In addition, this study focused on a Chinese Q&A site. The culture of cooperation may play crucial roles in information accumulation. Therefore, the study results may not be generalizable to sites in different languages or sites aimed at users from different cultures. Whether the effects of the marketplace of ideas on knowledge construction are generalizable to online knowledge-sharing platforms in other languages and countries warrants further research.

## Supplementary Information

Below is the link to the electronic supplementary material.

Supplementary information (DOCX 335 kB)

## Data Availability

The datasets used and analysed during the current study are available from the corresponding author on reasonable request.

## Footnotes

1 Sina Technology. (2018, December 13). Live|Zhuhu announces 220 million users, doubles growth in one year. https://tech.sina.com.cn/i/2018-12-13/doc-ihmutuec8882383.shtml

## Abbreviations

Q&A sites: Question-and-answer Websites
OKSPs: Online Knowledge Sharing Platforms
HSK: the graded vocabulary for Chinese Proficiency Test (HSK)
HLM: Hierarchical Linear Modeling
ICC: Intraclass Correlation Coefficient
