# Supplementary material for: Dynamics of the social construction of knowledge: an empirical study of Zhihu in China
Source: EPJ Data Sci. 2022 Jun 4;11(1):35. doi: 10.1140/epjds/s13688-022-00346-6 (PMC9166185; doi:10.1140/epjds/s13688-022-00346-6)
Supplement: Supplementary file 1 — Supplementary information (DOCX 335 kB) [file 13688_2022_346_MOESM1_ESM.docx]

**Appendix**

1. **Introduction of Zhihu.com**

As a Q&A site, Zhihu.com is an example of a large-scale platform for knowledge construction. Zhihu is designed to allow people to ask and answer questions on a broad range of topics. Similar to Quora and StackOverflow, questions on Zhihu are resolved by user-generated answers. The question–answer threads on Zhihu are well-defined and easily identifiable artifacts. A question–answer thread is a hierarchically organized collection of messages, with an initial answer to the original question and with subsequent messages written as answers to earlier messages. Users can also post comments and reply to one another under each answer. Figure 1 demonstrates the structure of the websites. Figure 2 is a sample page of Zhihu.com.


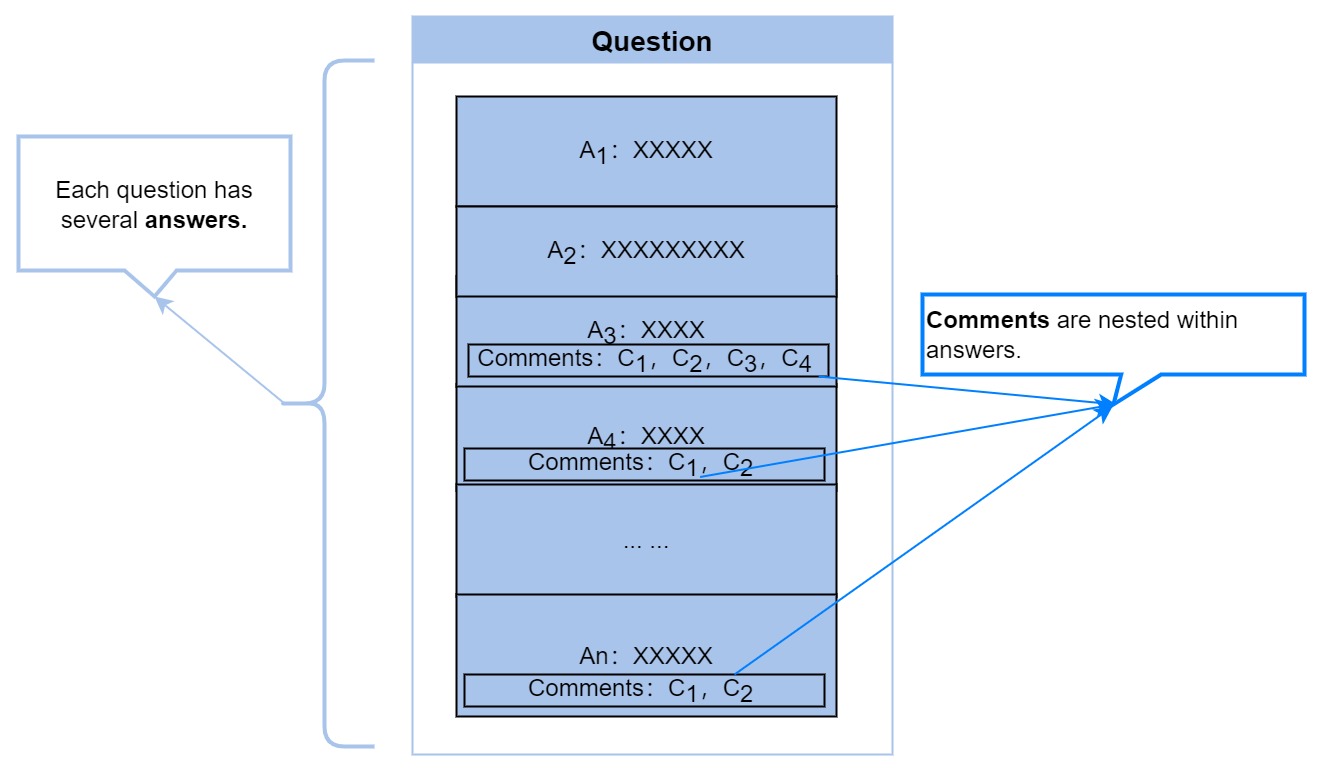


Figure 1 Demonstration of the structure of Zhihu.com


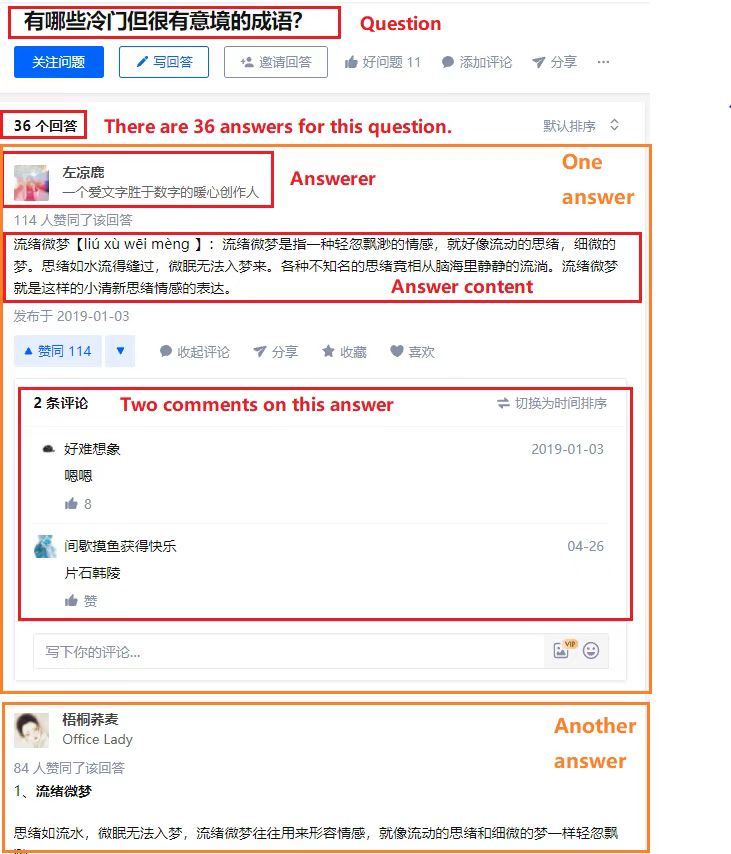


Figure 2 Sample page of Zhihu.com

1. **Measurement of readability**

Readability R corresponded to the combination of the average number of words per sentence and the average HSK grade of the vocabulary and was calculated as follows:

$\boldsymbol{R}=\ln\left( \frac{\boldsymbol{W}}{\boldsymbol{N}} \right)+\frac{\sum_{\boldsymbol{i}=\mathbf{1}}^{\boldsymbol{T}} \boldsymbol{Li}}{\boldsymbol{T}}$,

where W represents the word count of a given answer, N represents the total number of sentences, T represents the total number of words assigned an HSK grade, and Li represents the score of the ith word assigned an HSK grade (i ∈ [1, T]). We scored words from HSK levels 1 to 6 as 1 to 6, respectively (Li∈ [1, 6]). Readability is an inverse measure; the higher the readability score of a text, the higher the complexity of that text. Table 1 provides an example of calculating the readability score for answers.

Table 1 Example of Readability Score

|  | **Original Text** | **Translation** |
| --- | --- | --- |
| **Question** | 对于记忆力超群的孩子，家长应该如何培养？ | How should parents raise a child who has great memory? |
| **Answer** | 记忆力是学习能力的关键要素之一。记忆力强的孩子学习起来很轻松。新高考更注重学生兴趣和专业的结合，家长需要关注孩子的兴趣和特长。家长要让孩子的强记忆力在此领域上深度发展。 | Memory is one of the most critical elements of a child’s ability to learn. Children with great memory will learn quickly. The National College Entrance Examination will select students who apply their personal interests in a professional field. Therefore, parents should focus on balancing the personal interests and professions of their children. In other words, parents should develop their children’s memory skills in relation to the field in which their children are interested. |
| **Words retrieved after segmentation (tokenization and removing stopwords):** | 记忆力, 学习, 能力, 关键, 要, 记忆力, 孩子, 学习, 轻松, 高考, 注重, 学生, 兴趣, 专业, 结合, 家长, 关注, 孩子, 兴趣, 特长, 孩子, 记忆力, 领域, 发展 | memory, learn, ability, critical, element, memory, child, learn, quick, National College Entrance Examination, select, student, person interests, profession, apply, parent, focus, child, interest, profession, parent, child, memory, field, develop |
| **Words assigned an HSK grade** | 记忆力 (6) , 学习 (1) ,能力(3) ,记忆力 (6) ,孩子(1); 学习 (1) , 轻松(1) ,学生(1); 兴趣(3), 家长(2), 孩子(1), 兴趣(3), 孩子(1), 记忆力(6), 领域(3), 发展(2) | memory (6), learn (1), ability (3), memory (6), child (1), learning (1), quick (1), student(1)，interest(3), parent(2), child (1), interest(3), child (1), memory (6), field(3), develop(2), |
| **the word count of the given answer(W)** | 24 | |
| **Number of sentence(*N*)** | 4 | |
| $\ln\left( \frac{\boldsymbol{W}}{\boldsymbol{N}} \right)$ | 1.79 | |
| ***The* total number of words assigned an HSK grade(T)** | 16 | |
| **the score of the ith word that assigned an HSK grade (i ∈ [1, T])** $\sum_{\boldsymbol{i}\mathbf{=1}}^{\boldsymbol{T}} \boldsymbol{Li}$ | 6+1+3+6+1+1+1+1+3+2+1+3+1+6+3+2=41 | |
| $\frac{\sum_{\boldsymbol{i}\mathbf{=1}}^{\boldsymbol{T}} \boldsymbol{Li}}{\boldsymbol{T}}$ | 41/16=2.63 | |
| **Readability** | $\boldsymbol{R}=\ln\left( \frac{\boldsymbol{W}}{\boldsymbol{N}} \right)+\frac{\sum_{\boldsymbol{i}=\mathbf{1}}^{\boldsymbol{T}} \boldsymbol{Li}}{\boldsymbol{T}}$=1.79+2.56=4.35 | |

1. **Validation of measurement of information accumulation**

To further validate the measurement of information accumulation, we selected 953 questions with more than 20 answers to compare the distribution of information accumulation between real data and random-shuffled data. The number of answers is 84,787. Figure 3a is the distribution of information accumulation for real data. In comparison, we then randomly shuffled the sequence of the answers 100 times and demonstrated the distribution of information accumulation, as shown in Figure 3b. We then conducted a Kolmogorov–Smirnov test [1] to compare the distribution between the real data and randomly shuffled data. Two distributions were significantly different (D = 0.3164, p <.001). The results indicate that the measure of information accumulation did not decay by construction.

| 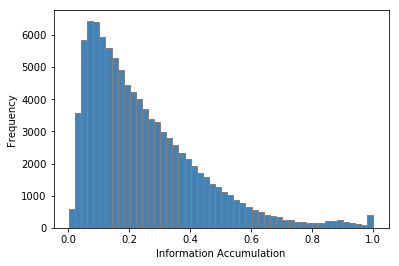 | 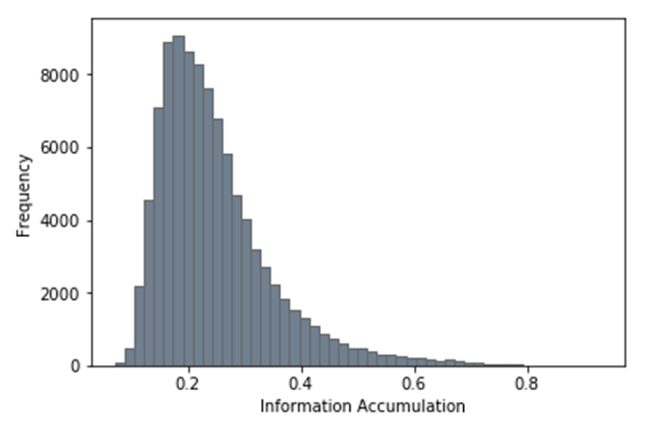 |
| --- | --- |
| Figure 3a. | Figure 3b. |
|  |  |

1. **HLM regression for the interaction effect of number of answers and Gini Coefficient**

To further investigate whether the effect of marketplace of ideas will vary across different number of answers, we examined the interaction effect of number of answers and Gini Coefficient on information accumulation. As shown in Table 2, it is found that the inclusion of number of answers (i.e., Model 3) did not substantially change the model. In addition, the negative effects of Gini coefficient on information accumulation (β = −.03, p < 0.001)) is robust across questions with different number of answers. As demonstrated in Figure 4 below, the effect of Gini coefficient of number of comments is stronger among questions with large number of comments.


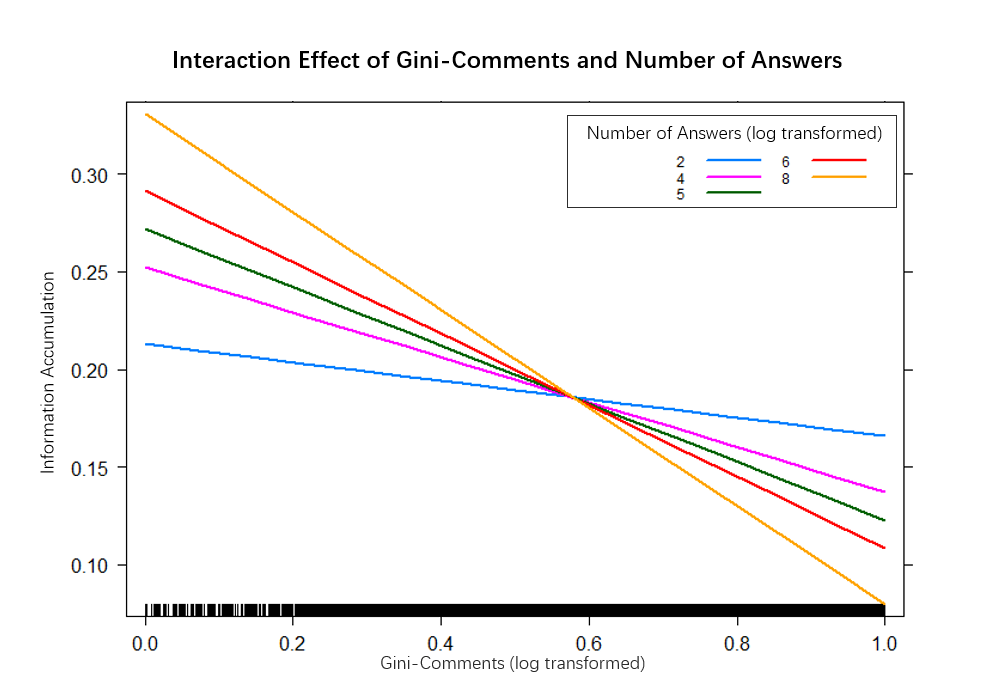


Figure 4 Interaction effect of the number of answers and Gini coefficient of number of comments on information accumulation

**Table 2 HLM regression for information accumulation**

|  | **Model 1** | | **Model 2** | | **Model 3** | |
| --- | --- | --- | --- | --- | --- | --- |
|  | **Estimates** | **Errors** | **Estimates** | **Errors** | **Estimates** | **Errors** |
| **Fixed Effects** |  |  |  |  |  |  |
| Intercept | .28 | .002 | .46 | .01 | .51 | .01 |
| ***Marketplace of Ideas*** |  |  |  |  |  |  |
| Gini-Answers Length |  |  | -.11^***^ | .01 | -.11^***^ | .01 |
| Gini-Comments |  |  | -.11^***^ | .01 | -.15^***^ | .01 |
| ***Participation Features*** |  |  |  |  |  |  |
| Presence of Active Users^a^ |  |  |  |  |  |  |
| Highly Active Users (top 1%) |  |  | -.01^***^ | .003 | -.01^***^ | .002 |
| Active Users (top 2-10%) |  |  | -.01^***^ | .002 | -.01^***^ | .002 |
| Anonymous |  |  | .001 | .001 | .001 | .001 |
| ***Temporal Features*** |  |  |  |  |  |  |
| Time-Interval between Answers^b^ |  |  | -.01^***^ | .004 | -.02^***^ | .004 |
| Order of Answers |  |  | -1.44^***^ | .08 | -1.41^***^ | .08 |
| ***Discourse Features*** |  |  |  |  |  |  |
| Sentiment |  |  | .0002 | .001 | .0002 | .001 |
| Readability^c^ |  |  | -.01^**^ | .003 | -.01^**^ | .003 |
| ***Control Variables*** |  |  |  |  |  |  |
| Question Type: Opinion^d,e^ |  |  | .003 | .004 | .003 | .004 |
| Question Type: Fact^d,e^ |  |  | -.04^***^ | .01 | -.04^***^ | .01 |
| Number of Answers per question ^b,d^ |  |  | -.004^*^ | .002 | -.01^***^ | .002 |
| Number of Answers * Gini-Comments |  |  |  |  | -.03^***^ | .01 |
| Number of Answers * Gini-Answers Length |  |  |  |  | -.002 | .005 |
| ***Random Effects*** |  |  |  |  |  |  |
| Intercept T_00_ | .07 | .16 | .07 | .15 | .07 | .15 |
| *Model Fit Statistics* |  |  |  |  |  |  |
| Log-Likelihood | 37451 | | 39705 | | 39726 | |

*Notes*. *** *p* < 0.001; ** *p* < 0.01; * *p* < 0.05
a: Highly active users are users whose answering frequencies rank in the top 1%. Active users

are users whose answering frequencies rank in top 2%–10%. Ordinary users are users whose answering frequencies rank in bottom 90%. The comparison group is ordinary users.

b: Variables are log transformed.

c: Readability is an inverse measure. The higher the readability score of a text is, the higher the

complexity of the text is.

d: Variables at level 2 (i.e., the question level).

e: The comparison group is Experience-based questions.

**References**

1. Berger VW, Zhou Y (2014) Kolmogorov–smirnov test: Overview. Wiley statsref: Statistics reference online
